# Impacts of completely endophytic renal masses on perioperative, oncologic, and functional outcomes in robot-assisted partial nephrectomy: a systematic review and meta-analysis

**DOI:** 10.3389/fonc.2024.1444477

**Published:** 2024-10-25

**Authors:** Han-xiao Gu, Jia Lv, Yi Liu, Hai-long Wang

**Affiliations:** ^1^ Department of Urology, Baoji Traditional Chinese Medicine Hospital, Baoji, China; ^2^ Department of Urology, The Second Hospital of Lanzhou University, Lanzhou, China

**Keywords:** completely endophytic tumors, non-completely endophytic tumors, robot-assisted partial nephrectomy, outcomes, meta-analysis

## Abstract

**Background:**

The objective of this study was to perform a comprehensive pooled analysis aimed at comparing the efficacy and safety of robot-assisted partial nephrectomy (RAPN) between completely endophytic tumors (CERT) and non-completely endophytic tumors (non-CERT).

**Methods:**

This study adhered rigorously to the Preferred Reporting Items for Systematic Reviews and Meta-Analyses (PRISMA) guidelines to conduct a systematic review and meta-analysis. We performed a systematic search in the PubMed, Embase, Web of Science, and Cochrane Library databases, focusing on studies published in English up to May 2024. Our analysis primarily evaluated key outcomes, specifically perioperative, functional, and oncological outcomes.

**Results:**

A total of 2126 patients across six studies were included in the analysis. Compared to non-CERT, CERT was associated with significantly higher rates of major complications (Odds Ratio [OR]: 2.47; 95% CI: 1.14 to 5.34; p = 0.02), longer warm ischemia times (Weighted Mean Difference [WMD]: 3.27 min; 95% CI: 0.61 to 5.39; p = 0.02), a greater decline in estimated glomerular filtration rate (eGFR) (WMD: 2.93 ml/min/1.73 m^2^; 95% CI: 0.75 to 5.11; p = 0.008), and relatively lower trifecta achievement rates (OR: 0.63; 95% CI: 0.41 to 0.96; p = 0.03). However, no statistically significant differences were observed between the two groups in terms of operative time, length of stay, blood loss, transfusion rates, intraoperative complications, overall complications, positive surgical margins, and local recurrence.

**Conclusions:**

Although CERT was associated with greater declines in eGFR and lower rates of trifecta achievement, it yielded perioperative, functional, and oncologic outcomes comparable to those of non-CERT in RAPN. Our findings suggest that RAPN for completely endophytic renal masses can achieve acceptable outcomes when performed in centers with substantial expertise in robotic surgery.

**Systematic review registration:**

https://www.crd.york.ac.uk/prospero/display_record.php?RecordID=555067, identifier CRD42024555067.

## 1 Introduction

Partial nephrectomy (PN) is widely recognized as the preferred therapeutic strategy for small renal tumors, in alignment with recommendations from the American Urological Association (AUA) and the European Association of Urology (EAU) guidelines (1, 2). Beyond yielding surgical outcomes and cancer control comparable to those of radical nephrectomy, PN offers the distinct advantage of nephron preservation. This preservation is pivotal not only for maintaining renal function but also for enhancing postoperative quality of life in patients (3). In recent years, the advent of robotic technology has revolutionized the field of PN, leading to substantial advancements in both instrumentation and surgical techniques. As a result, robot-assisted partial nephrectomy (RAPN) has gained ascendancy over traditional laparoscopic PN. This shift is characterized by significant enhancements in perioperative outcomes and a marked reduction in the learning curve, making RAPN an increasingly favored approach in urological surgery (4–6).

The Complete Endophytic Renal Tumor (CERT) is typically evaluated using the ‘E’ domain of the RENAL Nephrometry Score, which assesses the extent of tumor invasion into the normal renal parenchyma (7). Tumors are classified into three groups based on their growth patterns: exophytic, mesophytic, and endophytic. Moreover, many surgeons contend that the complexity of tumors, particularly those that are entirely endophytic, substantially increases the difficulty of surgical procedures. These complex tumors pose numerous challenges for the surgeon, requiring advanced skills and careful planning (8). Despite previous studies indicating that RAPN can be safely performed even on completely endophytic tumors (9), a significant barrier to drawing definitive conclusions is the reliance on research characterized by small sample sizes and conducted within the confines of single institutions. These limitations hinder the ability to achieve robust and universally applicable results, calling for broader multi-institutional studies to validate these findings.

Therefore, the objective of this study is to synthesize comparative research data to evaluate the efficacy and safety of RAPN for CERT versus non-CERT. This research aims to provide a comprehensive analysis of the available evidence, thereby informing and guiding clinical decision-making processes.

## 2 Methods

This study was officially registered with PROSPERO and adhered meticulously to the Preferred Reporting Items for Systematic Reviews and Meta-Analyses (PRISMA) guidelines, in accordance with the recommendations of the 2020 statement (10, 11). Additionally, it has been documented in the PROSPERO registry under the identification number: CRD42024555067.

### 2.1 Literature search strategy, study selection, and data collection

We conducted exhaustive searches across multiple databases including PubMed, Embase, Web of Science, and the Cochrane Library, capturing data up to May 2024. Our search methodology employed a combination of terms specific to the intervention and relevant to patient characteristics, structured as follows: [(Robotic PN OR Robot-assisted PN OR Robot-assisted nephron-sparing surgery) AND (Intrarenal OR Endophytic OR Completely endophytic) AND (Renal tumors OR Renal masses)]. Additionally, we conducted manual searches of relevant references to ensure thoroughness and broaden the scope of our investigation.

The inclusion criteria were established utilizing the PICOS framework: P (Patients)—included patients diagnosed with localized renal tumors; I (Intervention)—patients with CERT who underwent RAPN; C (Comparator)—patients diagnosed with non-CERT, also treated with RAPN; O (Outcome)—evaluated outcomes encompassed perioperative metrics, complications, renal functionality, and oncologic effectiveness; S (Study Type)—the studies considered were randomized controlled trials (RCTs), along with prospective and retrospective comparative studies. Exclusion criteria were delineated as follows: (1) specific types of publications, such as case reports, meeting abstracts, editorial comments, and any unpublished research; (2) studies lacking crucial data required for inclusion in a meta-analysis; (3) studies that failed to provide comparative data.

Each selected study was meticulously reviewed by two independent evaluators. The extracted data included: (1) General study details such as the first author, year of publication, and country of the study; (2) Participant demographics, which covered sample size, age, gender, body mass index (BMI), RENAL scores, and follow-up duration; (3) Perioperative outcomes, including operative time, hospital stay duration, warm ischemia time, blood loss, intraoperative complications, major complications (Clavien grade ≥ 3), and overall complications (Clavien grade ≥ 1) (12); (4) Renal function and oncologic outcomes, encompassing preoperative estimated glomerular filtration rate (eGFR), trifecta achievement, tumor diameter and site, clinical stage, tumor pathology, local recurrence, and positive surgical margins (PSM). All discrepancies were resolved through consensus or following consultation with a third reviewer.

This study employed the Risk of Bias in Non-randomized Studies of Interventions (ROBINS-I) framework to evaluate non-RCTs (13). The quality of the literature was independently assessed by two evaluators. Discrepancies in the evaluations were resolved through detailed discussion between the evaluators.

### 2.2 Statistical analysis

For data analysis, we utilized the RevMan5.4 software provided by the Cochrane Collaboration (Oxford, UK). Odds ratios (ORs) and weighted mean differences (WMD) for dichotomous and continuous variables were calculated separately, with results presented including a 95% confidence interval (CI). To determine heterogeneity among the included studies, the I^2^ test was applied (14). In light of expected significant heterogeneity, a random-effects model was adopted for all statistical analyses, with a p-value of less than 0.05 indicating statistical significance. Sensitivity analyses were also conducted on results with marked heterogeneity to explore the sources of such variability between studies and to verify the robustness of our analyses.

### 2.3 Publication bias

In our study, we employed Begg’s funnel plot method to systematically assess and identify potential evidence of publication bias.

## 3 Results

### 3.1 Baseline characteristics

**Figure 1:**
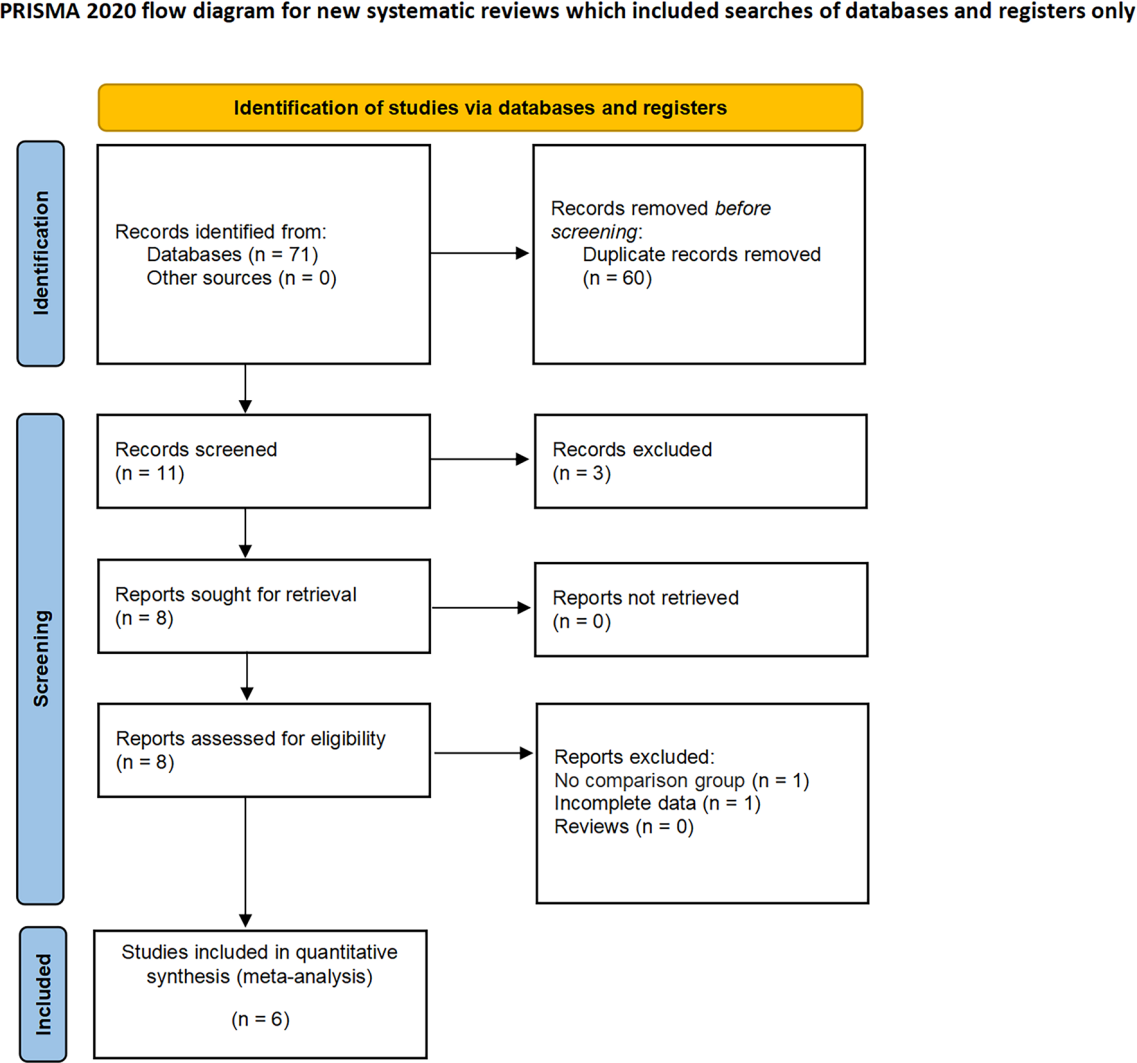
PRISMA flow diagram for the systematic review.

In our systematic review, we initially identified 71 relevant studies. After the removal of duplicates, 11 studies remained for detailed assessment. Further screening of titles and abstracts led to the exclusion of three studies, as they were not controlled studies, and an additional two were discarded following an exhaustive full-text review. Consequently, our meta-analysis ultimately included six studies, involving a total of 2,126 patients, comprising 389 diagnosed with CERT and 1,737 with non-CERT conditions, as illustrated in **Figure 1**. The cohort for analysis consisted of six non-RCTs, all of which were retrospective comparative studies (15–20). Of these, two studies were multi-institutional (15, 17), while the others were conducted at single centers. The scope of the research was international, with studies originating from Japan, the United States, Europe, and Korea. None of the included studies used propensity scoring analysis. **Table 1** offers a meticulous summary of the key characteristics of the studies, including preoperative variables and interventions—detailing sample size, age, gender, and BMI. The duration of follow-up for the studies included ranged from 12 to 48 months. **Tables 2**, **3** provide an exhaustive summary of the functional and oncological outcomes.

**Table 1:** The trials included in the systemic review.

Reference	Year	Country	Propensity scoring analysis	Center	Patients		Age(y)		Male/Female		BMI (kg/m^2^)	
					Completely endophytic	Non-completely endophytic	Completely endophytic	Non-completely endophytic	Completely endophytic	Non-completely endophytic	Completely endophytic	Non-completely endophytic
Ito	2024	Japan	No	multi-institutional	76	590	62.2(12.8)	62.7(12.9)	63/13	412/178	24.5(3.6)	24.5(4.3)
Motoyama	2022	Japan	No	single-center	26	127	64.5(12.5)	68(15.75)	18/8	83/44	24.8(5.43)	24.2(8.05)
Carbonara	2020	USA and Europe	No	multi-institutional	147	510	57.7(11.8)	60.9(12.7)	93/54	296/214	27.4(5.7)	27.7(5.2)
Curtiss	2015	USA	No	single-center	30	267	54.5(10)	60.9(10.4)	14/16	170/97	30.2(6.7)	29.4(5.9)
Komninos	2014	Korea	No	single-center	45	64	50(9.63)	51(10.37)	31/14	30/34	26.1(3.33)	25.5(3.7)
Autorino	2014	USA	No	single-center	65	179	56(1.4)	61.2(0.9)	31/34	111/68	29.4(6.3)	31.2(7.4)

BMI, Body mass index; Mean (SD).

**Table 2:** The trials included in the systemic review.

Reference	Tumor diameter (cm)		Tumor site (Lt/Rt)		Preoperative eGFR (ml/min/1.73 m^2^)		RENAL score		Follow-up duration (month)	
	Completely endophytic	Non-completely endophytic	Completely endophytic	Non-completely endophytic	Completely endophytic	Non-completely endophytic	Completely endophytic	Non-completely endophytic	Completely endophytic	Non-completely endophytic
Ito	2.4(0.84)	2.9(1.3)	NA		71.6(16.2)	68.6(19.1)	8.9(1.3)	6.8(1.7)	12(46.7)	12.5(62.2)
Motoyama	1.9(1.0)	2.9(1.75)	8/18	63/64	NA		9(1.25)	6(1.5)	NA	
Carbonara	4.2(2.5)	3.2(4.1)	NA		84.2(22.7)	83.6(21.4)	10(1.48)	4(1.48)	21.6(20)	32.3(25.4)
Curtiss	2.3(1.1)	2.7(1.4)	NA		NA		9(1.5)	6(2.2)	10.6	
Komninos	2.6(1.56)	2.5(2.96)	26/19	29/35	84.4(10.37)	90(14.07)	9(1.48)	5.5(2.22)	48(28.89)	38(34.82)
Autorino	2.6(1.0)	3.7(2.1)	32/33	90/89	89.6(22.9)	80.1(23.2)	8.7(1.4)	6.4(2.2)	12.6(11.0)	14.5(13.8)

eGFR, estimated glomerular filtration rate; Mean (SD).

N/A, not application.

**Table 3:** Oncologic outcomes.

Reference	Tumor stage		Tumor pathology	
	Completely endophytic	Non-completely endophytic	Completely endophytic	Non-completely endophytic
Ito	pT1a:62; pT1b:1; pT2a:0; pT3a:5	pT1a:438; pT1b:68; pT2a:1; pT3a:31	Clear cell: 54; Papillary: 3; Chromophobe: 6; Others: 13	Clear cell: 426; Papillary: 48; Chromophobe: 37; Others: 79
Motoyama	NA		Clear cell: 18; Others: 2; Benign: 6	Clear cell: 75; Others: 24; Benign: 28
Carbonara	pT1a:68; pT1b:33; pT2a:13; pT2b:2; pT3a:10	pT1a:307; pT1b:70; pT2a:9; pT2b:4; pT3a:18	Benign: 31; Malignant: 116	Benign: 121; Malignant: 389
Curtiss	pT1a:19; pT1b:0; pT2a:0; pT3a:1	pT1a:161; pT1b:31; pT2a:3; pT3a:31	Clear cell: 15; Papillary: 2; Chromophobe: 0; Others: 3	Clear cell: 110; Papillary: 45; Chromophobe: 20; Others: 33
Komninos	pT1a:30; pT1b:9; pT2:1; pT3a:0	pT1a:30; pT1b:10; pT2:4; pT3a:2	Benign: 5; Malignant: 40	Benign: 18; Malignant: 46
Autorino	pT1a:47; pT1b:3; pT2:0; pT3a:2	pT1a:84; pT1b:41; pT2:4; pT3a:11	Benign: 17; Malignant: 48	Benign: 40; Malignant: 139

N/A, not application.

The analysis demonstrated that individuals within the CERT group were on average younger than those in the non-CERT group, with a WMD of -3.48 years (95% CI: -5.39 to -1.57; p = 0.0004). Additionally, patients in the CERT group presented with higher RENAL scores compared to the non-CERT group, evidenced by a WMD of 3.32 (95% CI: 1.72 to 4.92; p < 0.0001). However, comparative analyses regarding BMI, tumor diameter, and preoperative eGFR between the two groups did not exhibit any statistically significant differences, with p-values of 0.36, 0.18, and 0.58, respectively, as detailed in **Table 4**.

**Table 4:** Comparison of baseline characteristics of patients.

Baseline characteristic	CERT VS non-CERT group	Heterogeneity I^2^ (%)	*p* value
Age WMD (95% CI)	-3.48(-5.39 to -1.57)	71	0.0004
BMI WMD (95% CI)	-0.24(-0.77 to 0.28)	0	0.36
Tumor diameter WMD (95% CI)	-0.35(-0.87 to 0.17)	89	0.18
RENAL score WMD (95% CI)	3.32(1.72 to 4.92)	99	< 0.0001
Preoperative eGFR WMD (95% CI)	1.56(-3.73 to 6.85)	81	0.56

CERT, completely endophytic tumors; non-CERT, non-completely endophytic tumors; eGFR, estimated glomerular filtration rate.

### 3.2 Assessment of quality

All studies included in the analysis conducted comparative evaluations, with the majority being published between 2014 and 2024. An assessment of the risk of bias indicated that five studies were categorized as having a moderate risk, while one exhibited a high risk of bias (18). These assessments are comprehensively detailed in **Supplementary Table 1**.

### 3.3 Outcome analysis

#### 3.3.1 Perioperative effectiveness

**Figure 2:**
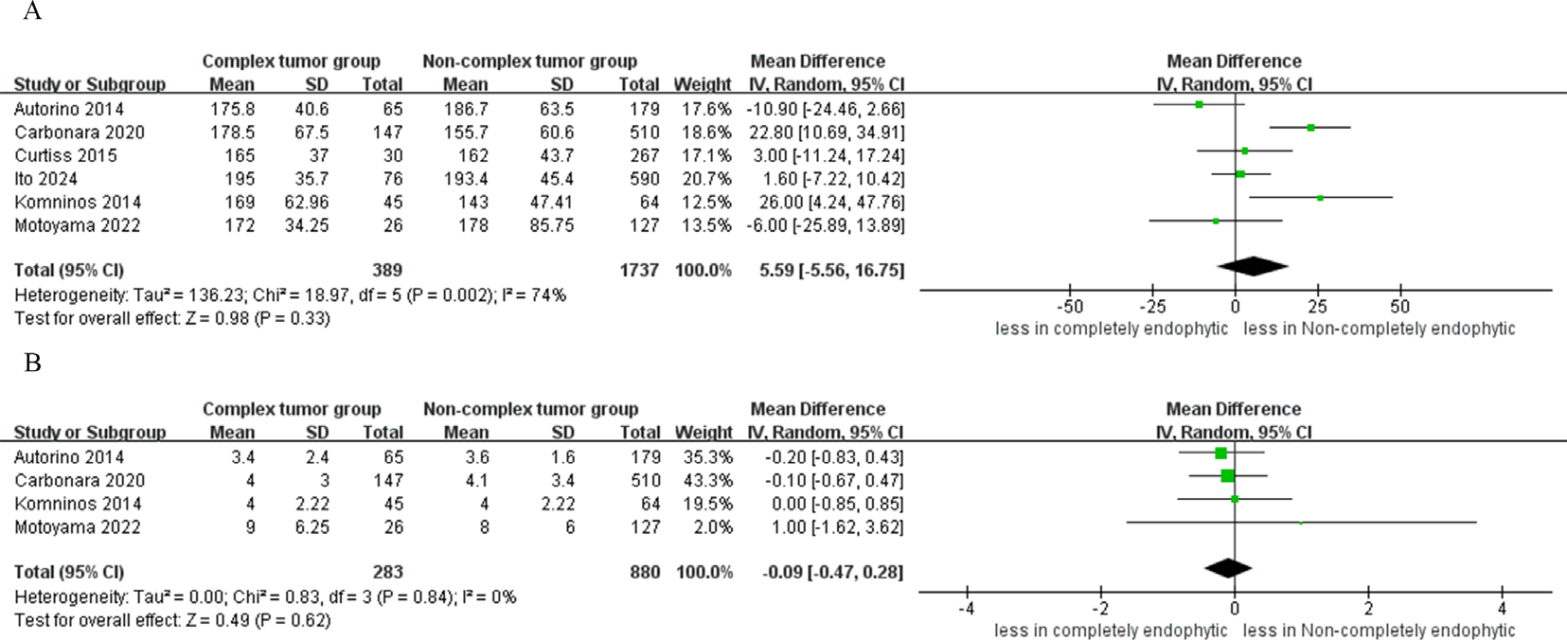
Forest plots of perioperative outcomes **(A)** operative time, **(B)** length of hospital stay.

The pooled results from six studies indicated no significant difference in operative time between the CERT and non-CERT groups (WMD 5.99 min, 95% CI -5.56 to 16.75; p = 0.33) (15–20). Additionally, the meta-analysis, which included four studies, reported that the cumulative findings showed no significant differences in the length of hospital stay between the two groups (WMD -0.09 day, 95% CI -0.47 to 0.28; p = 0.62) (16, 17, 19, 20), as depicted in **Figure 2**.

**Figure 3:**
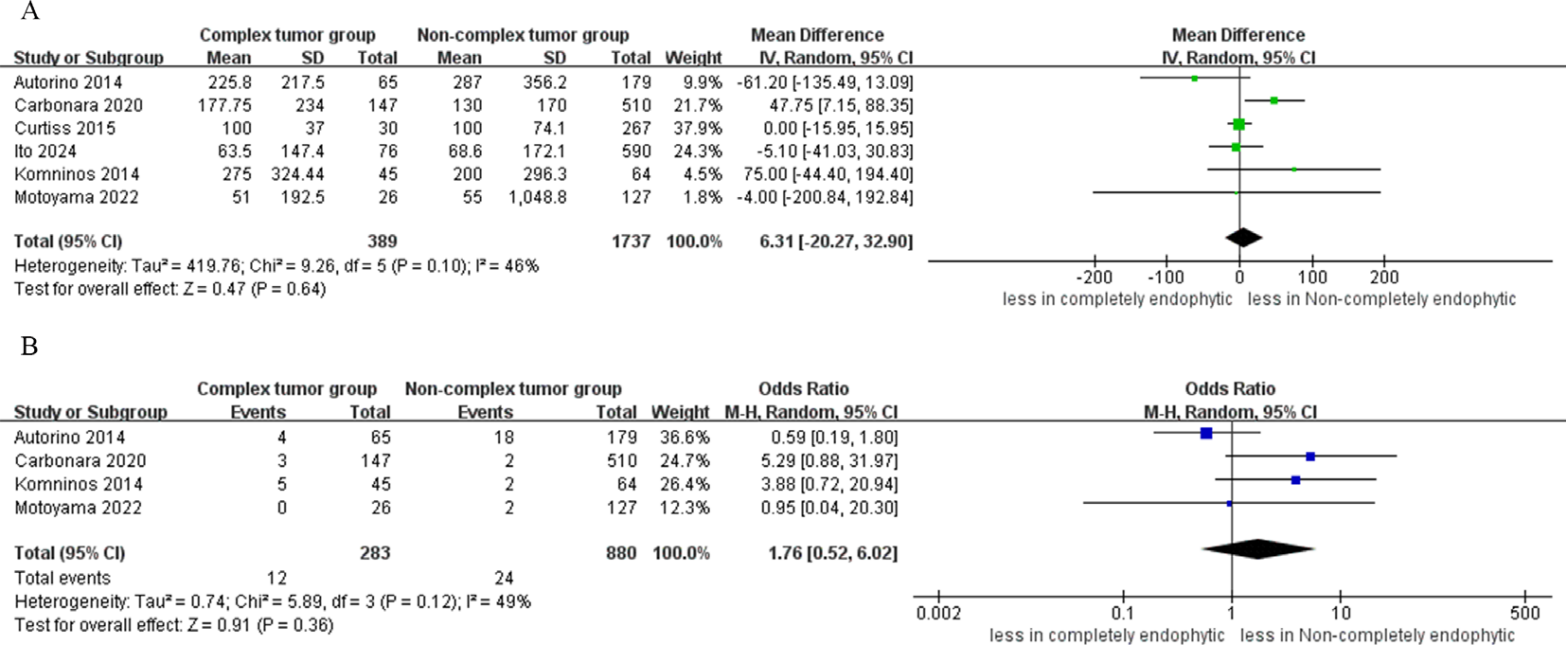
Forest plots of perioperative outcomes **(A)** blood loss, **(B)** transfusion rates.

The analysis revealed no statistically significant differences in blood loss between the CERT and non-CERT tumor groups (six studies; WMD 6.31 ml, 95% CI -20.27 to 32.90; p = 0.64) (15–20). Similarly, cumulative analysis showed no significant differences in transfusion rates between the two groups, based on data from four studies (OR: 1.76; 95% CI: 0.52 to 6.02; p = 0.36) (16, 17, 19, 20), as depicted in **Figure 3**.

#### 3.3.2 Complications

**Figure 4:**
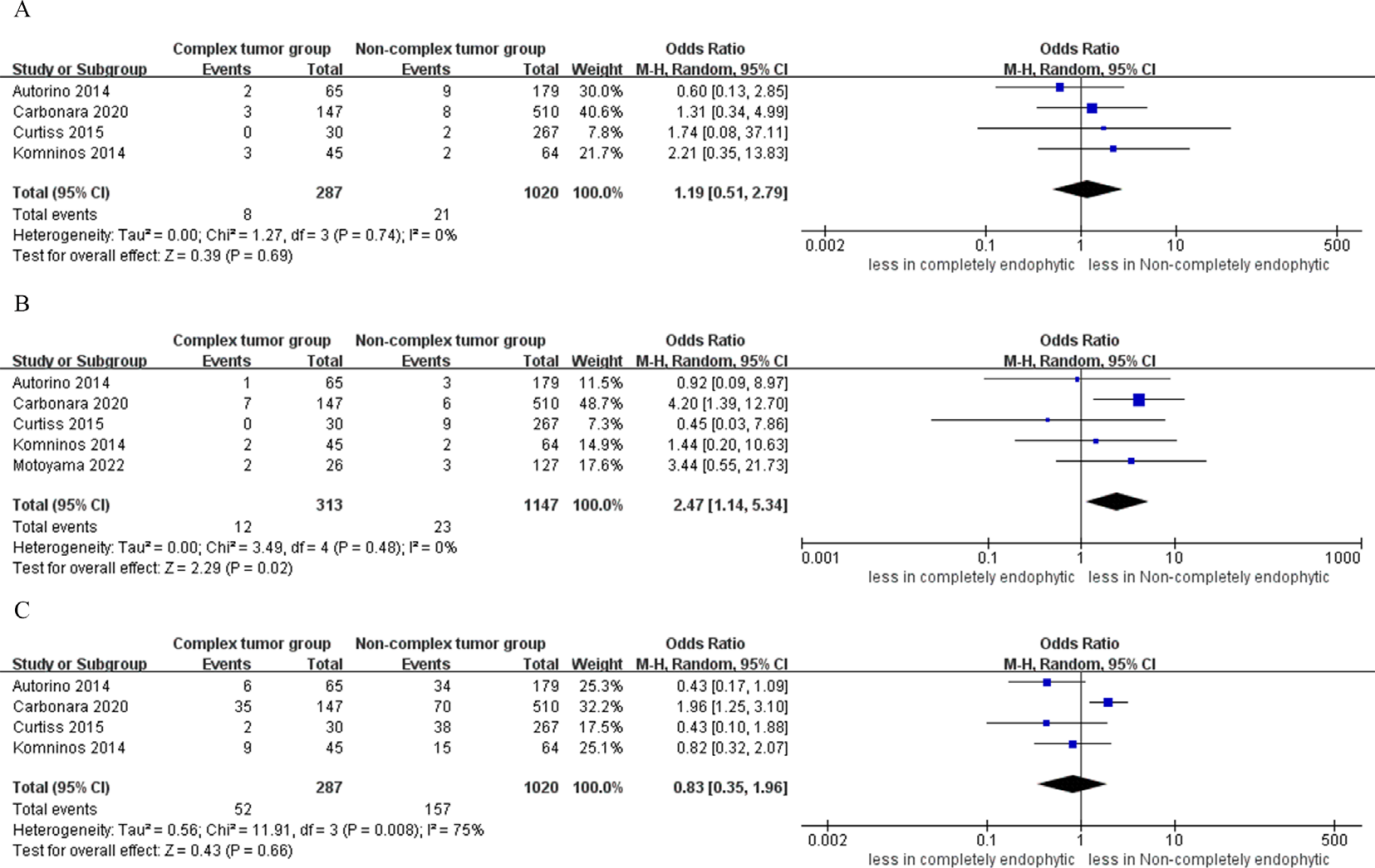
Forest plots of complication **(A)** intraoperative complications, **(B)** major complication **(C)** overall complications.

Analysis from four studies indicated that the difference in the incidence of intraoperative complications between the CERT and non-CERT cohorts was not statistically significant (OR: 1.19; 95% CI: 0.51 to 2.79; p = 0.69) (17–20). In the CERT cohort, major complications occurred in 3.8% of cases (12 out of 313), whereas in the non-CERT cohort, the rate was 2.0% (23 out of 1147). However, the analysis revealed a statistically significant higher risk of major complications in the CERT group compared to the non-CERT group (OR: 2.47; 95% CI: 1.14 to 5.34; p = 0.02) (16–20). Furthermore, our meta-analysis of four studies that focused on overall complication rates showed that the CERT group experienced a complication rate of 18.1% (52 out of 287 cases), while the non-CERT group experienced a rate of 15.4% (157 out of 1020 cases). Nonetheless, the difference in overall complication rates between the CERT and non-CERT cohorts was not statistically significant (OR: 0.83; 95% CI: 0.35 to 1.96; p = 0.66) (17–20), as shown in **Figure 4**.

#### 3.3.3 Renal functional

**Figure 5:**
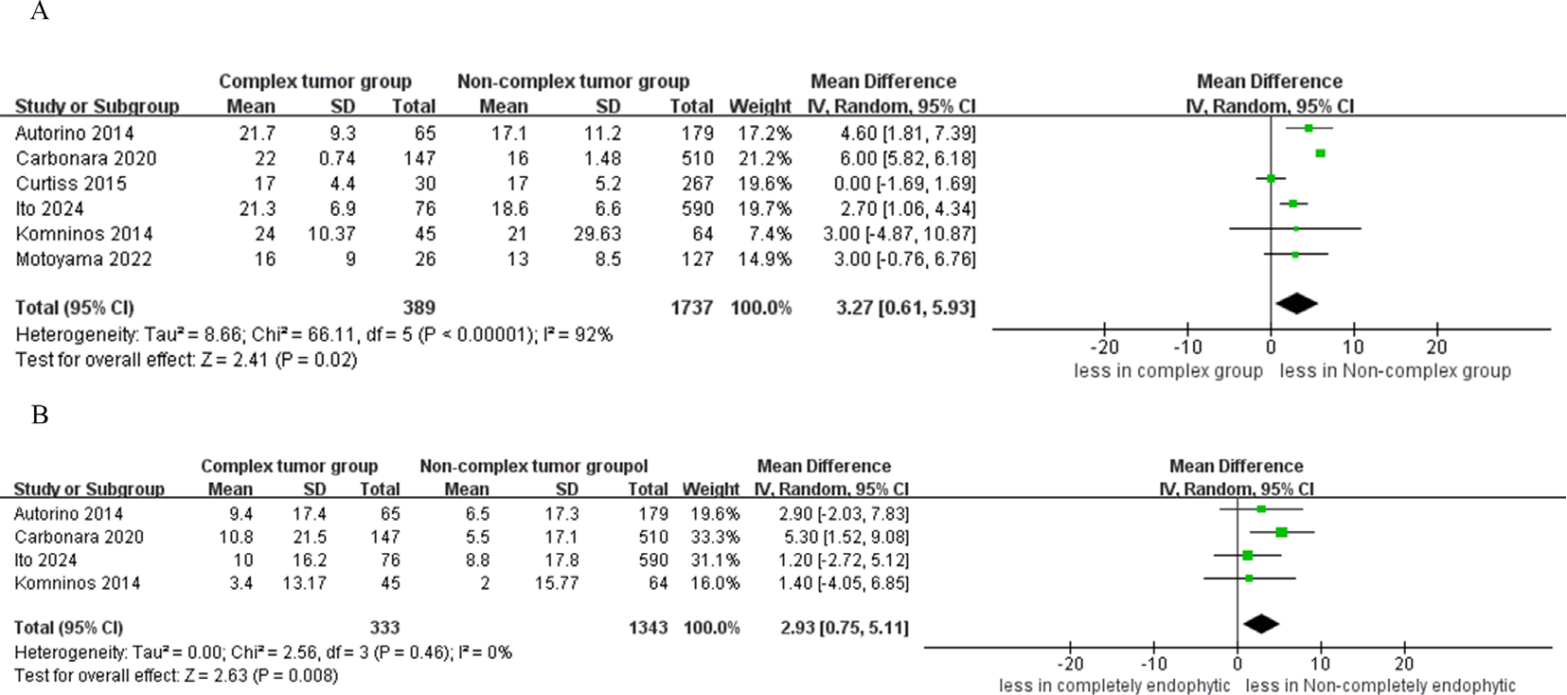
Forest plots of renal functional outcomes **(A)** warm ischemia time, **(B)** eGFR decline.

The quantitative analysis of six studies focused on warm ischemia time revealed that the CERT group experienced longer warm ischemia durations compared to the non-CERT group (WMD 3.27 min, 95% CI 0.61, 5.93; p = 0.02) (15–20). Additionally, a subsequent meta-analysis, which included data from four studies, indicated a greater decline in eGFR in the CERT group (WMD 2.93 ml/min/1.73 m^2^, 95% CI 0.75 to 5.11; p = 0.008) (15, 17, 19, 20), as depicted in **Figure 5**.

#### 3.3.4 Oncologic outcomes

**Figure 6:**
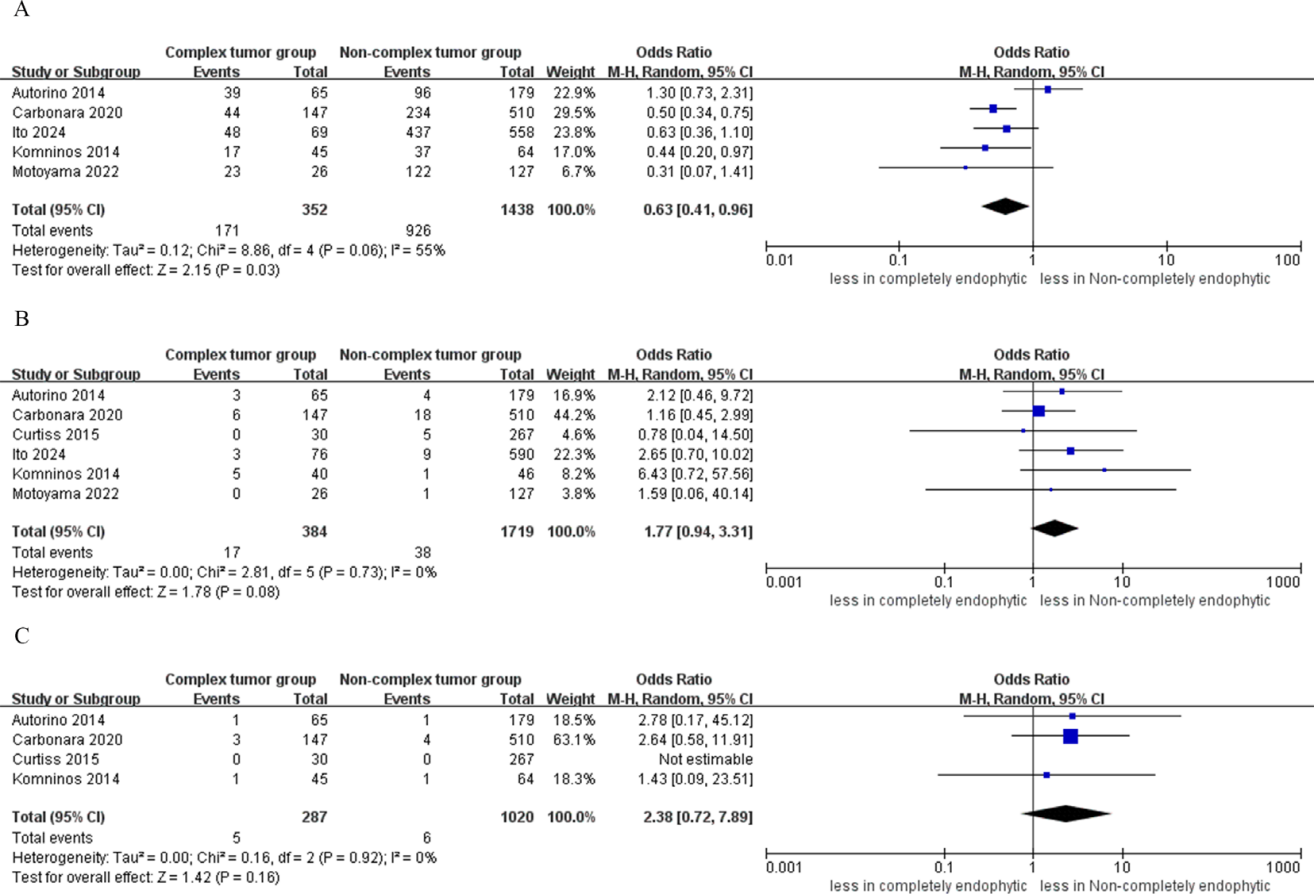
Forest plots of oncologic outcomes **(A)** trifecta achievement, **(B)** PSM, **(C)** local recurrence.

In the CERT group, the analysis indicated statistically significantly lower rates of trifecta achievement compared to the non-CERT group (five studies; OR 0.63, 95% CI 0.41 to 0.96; p = 0.03) (15–17, 19, 20). However, the analysis found no statistical significance in PSM between CERT and non-CERT across six studies (OR 1.77, 95% CI 0.94 to 3.31; p = 0.08) (15–20). Regarding local recurrence, the CERT group reported a rate of 1.7% (5 incidents out of 287 cases), while the non-CERT cohort had a rate of 0.5% (6 incidents out of 1020 cases). A meta-analysis of four studies showed that there was no statistically significant difference in local recurrence rates between the CERT and non-CERT groups across six studies (OR 2.38, 95% CI 0.72 to 7.89; p = 0.16) (17–20), as illustrated in **Figure 6**.

### 3.4 Heterogeneity

Our research findings generally demonstrate moderate heterogeneity. Despite including studies of moderate to high quality, we observed considerable heterogeneity in three outcomes: operative time (I² = 74%), overall complications (I² = 75%), and warm ischemia time (I² = 92%).

### 3.5 Sensitivity analysis

In this investigation, we noted significant heterogeneity across three clinical parameters: operative time, overall complications, and warm ischemia time. To pinpoint the primary sources of this heterogeneity and assess the robustness of our findings, we conducted a sensitivity analysis by systematically excluding one study at a time. It’s important to highlight that for outcomes where the number of included studies was three or fewer, sensitivity analyses were deemed inapplicable. Ultimately, this process did not reveal any substantial changes in the levels of heterogeneity associated with operative time, overall complications, and warm ischemia time. This suggests that the observed heterogeneity is a consistent characteristic across the included studies.

### 3.6 Publication bias

**Figure 7:**
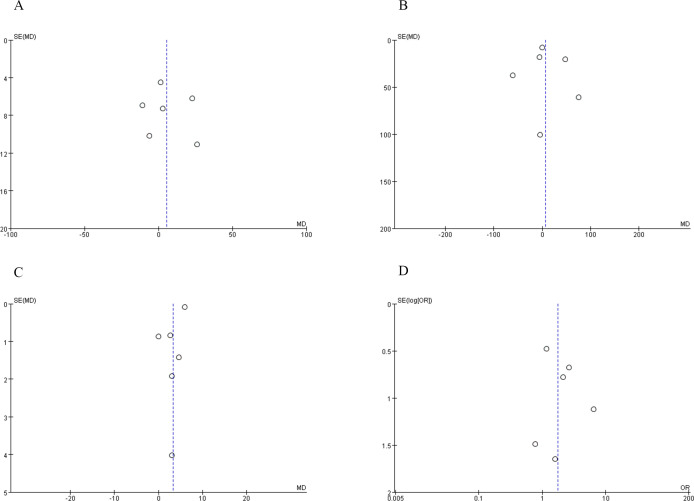
Funnel plot **(A)** operative time, **(B)** blood loss, **(C)** warm ischemia time, **(D)** PSM.

To evaluate the presence of publication bias, we analyzed indicators such as operative time, blood loss, warm ischemia time, and PSM. The distribution of studies exhibited near symmetry for these variables, suggesting a minimal likelihood of publication bias. These findings are detailed in **Figure 7**.

## 4 Discussion

This study aims to evaluate the perioperative, functional, and oncologic outcomes of RAPN for CERT and non-CERT. Additionally, several significant findings from this study warrant further discussion.

Due to the larger and deeper resection of normal renal parenchyma surrounding the tumor, a longer surgical time is typically required to completely remove an endophytic tumor. However, there was no statistically significant difference in surgical time between the two groups. Besides tumor characteristics, numerous other factors can influence surgical time, such as the experience of the surgeon and assistant, the patient’s BMI, and intraoperative complications (21). In the included studies, all procedures were performed by operators with extensive experience in minimally invasive surgery, which may partially explain this result. In most of the included studies, the average hospital stay for patients was 4 days. Robotic surgery helps to reduce intraoperative blood loss, maintain a clear surgical field, and protect surrounding tissues (22). Additionally, minimally invasive surgery aids in the recovery of bowel function and reduces complications associated with prolonged bed rest, thereby shortening the hospital stay. There was no statistically significant difference in hospital stay between the two groups. However, the hospital stay for robotic surgery is mainly influenced by the surgeon’s expertise and the volume of procedures at the institution, rather than the surgical method itself (23). It is also important to note that differences in healthcare systems and insurance policies across regions may lead to variations in hospital stay (24).

The combined results indicated no statistically significant difference in blood loss between the CERT and non-CERT groups (p = 0.64). Despite this, the CERT group generally exhibited greater blood loss across most included studies. This lack of statistical significance may be due to the limited number of studies analyzed. However, the increased blood loss in the CERT group was not likely to be clinically significant, as there was no significant difference in transfusion rates between the two groups (p = 0.36). The transfusion rates observed in both the CERT and non-CERT groups may also be influenced by the surgeon’s expertise and the hospital’s blood transfusion guidelines (25).

The analysis revealed that the CERT group exhibited a higher incidence of major complications compared to the non-CERT group (p=0.02). This finding may be attributed to the increased complexity of RAPN in tumor reconstruction and resection. It is noteworthy that no patients succumbed to major complications. Furthermore, cumulative analysis indicated no significant differences in intraoperative (p = 0.69) and overall complications between the two groups (p = 0.66). Therefore, despite the increased incidence of major complications in the CERT group, RAPN can still yield acceptable outcomes. In greater detail, two studies reported on the intraoperative conversion to radical nephrectomy in both patient groups, finding no significant differences in conversion rates between them. Similarly, one study noted that there were no cases requiring embolization for hemorrhage in either group (15, 18). However, more evidence is required to validate these conclusions.

For completely endophytic renal tumors, RAPN poses significant challenges in tumor localization and excision, leading to prolonged warm ischemia time. A quantitative analysis of five studies focusing on warm ischemia time revealed that the CERT group experienced longer warm ischemia durations compared to the non-CERT group (p = 0.02). However, certain aspects warrant attention, particularly the optimal time of warm ischemia during PN, which remains a topic of debate in the urological community. Several studies suggest that warm ischemia time should be limited to 25 or 30 minutes to minimize the risk of renal function impairment (26–28). It is noteworthy that the warm ischemia times included in our analysis were all less than 30 minutes. Considering these factors, the ischemia time in the CERT group is deemed acceptable. Postoperative renal function is crucial, especially for endophytic tumors (18). The meta-analysis, which included data from four studies, indicated a greater decline in eGFR in the CERT group. A meta-analysis including data from four studies indicates that the CERT group experienced a greater decline in eGFR. However, certain aspects deserve attention. First, recent studies suggest that preoperative renal function and the number of kidneys preserved are major factors significantly associated with long-term renal function outcomes (29, 30). Second, the work of Fergany et al. (31) highlights the critical role of age in the postoperative recovery of renal function. Additionally, the included studies did not report the number of patients who progressed to advanced stages of CKD during follow-up. Nonetheless, this result may not translate into clinical harm for patients. Therefore, this result should be interpreted with caution.

In our study, the trifecta achievement rate in the CERT group for treating CERT was lower at 48.6% (171 out of 352 cases) compared to reports on small renal masses in RAPN series (32). Factors influencing trifecta achievement include tumor size and complexity, with patients in the CERT group presenting higher RENAL scores than those in the non-CERT group, making these findings expected. Additionally, our results are consistent with those published by Bertolo et al. (33), who reported a trifecta achievement rates of 49% among patients with larger renal tumors treated with RAPN. The prolonged warm ischemia time in the complex tumor group appears to be a contributing factor affecting trifecta achievement. Nonetheless, trifecta achievement does not assess long-term renal function and oncological outcomes, indicating the necessity for further long-term follow-up studies to evaluate these results comprehensively. Among the included studies, no significant difference in PSM was observed between the CERT and non-CERT groups. The incidence of PSM in the CERT group was 4.42%, compared to 2.21% in the non-CERT group. The PSM rate of 4.42% in the CERT group aligns with the range reported by high-volume institutions performing RAPN, where rates vary from 0% to 3.7% (34). Several important aspects of this finding merit further discussion. Firstly, Marszalek et al. (35) suggested that PSM might not be a decisive factor for recurrence. Secondly, various factors could influence PSM, including tumor staging, surgical approach (transperitoneal or retroperitoneal approaches), and tumor diameter (36). Consequently, further research is essential to validate our findings. Furthermore, our study showed that there was no statistically significant difference in local recurrence rates between the CERT and non-CERT groups across six studies.

Other important issues requiring in-depth discussion include the choice of surgical approach. First, the studies we included utilized different surgical approaches, such as transperitoneal or retroperitoneal approaches. The retroperitoneal approach offers certain benefits; for example, it may result in shorter operative times and shorter hospital stays, particularly for posteriorly located tumors (37). However, compared to the transperitoneal approach, the retroperitoneal approach also has drawbacks, such as limited working space. The debate over whether to choose the retroperitoneal or transperitoneal approach remains controversial. Therefore, further research with higher-quality evidence is necessary to determine the most suitable surgical method for CERT. Second, three-dimensional (3D) virtual models have shown a positive impact. Grosso et al. (38) conducted a study reporting that 3D virtual models are promising tools, as they can provide a reliable assessment of surgical planning. However, with increasing complexity of the renal masses, the advantages offered by 3D reconstruction become more apparent. Additionally, another study reported that the use of 3D virtual models in RAPN resulted in a lower incidence of global ischemia and a higher enucleation rate compared to the control group (39). Therefore, the importance of surgical planning is crucial for RAPN for complete endophytic renal masses. Third, recent studies have compared the outcomes of open and robotic PN (enucleation, enucleoresection, or resection), focusing on predictors of trifecta failure in patients with highly complex renal tumors (40, 41). These studies have shown that tumor complexity and surgical approach are independent predictors of trifecta failure following PN for highly complex renal tumors. Fourth, the endophytic renal masses are only one determinant of tumor complexity. The complexity of renal tumors primarily depends on several tumor-associated factors, such as tumor size and type, including endophytic, hilar, and cystic renal tumors (42). Additionally, the RENAL and PADUA scores are among the most commonly used renal scoring systems, with complex renal tumors identified as those having a RENAL or PADUA score of 7 or higher (7, 43). Lastly, RAPN is a challenging surgical procedure that requires continuous learning and adaptation, influenced by various patient, tumor, and surgeon-related factors. Beyond the complexity of the tumor and the increasing volume of cases managed by surgeons, prior surgical experience significantly impacts perioperative outcomes (44). Incorporating research from different institutions may introduce some heterogeneity in the results. Therefore, more research is needed to confirm our conclusion.

The limitations of this study must be acknowledged. Firstly, all included studies were non-randomized controlled trials, inherently carrying a risk of potential bias. Secondly, the absence of subgroup analyses based on surgical approaches (retroperitoneal versus transperitoneal) in the included studies may have introduced subtle differences in outcomes. Thirdly, the lack of reported oncological outcomes such as cancer-specific survival (CSS), overall survival (OS), and recurrence-free survival (RFS) results in insufficient data for a comprehensive evaluation of oncological results. Fourth, the relatively short follow-up periods (10-12 months) in some studies constrain the ability to compare renal function and oncological outcomes between the two groups effectively. Finally, endophyticity may include different grades according to the amount of parenchyma above the lesion. However, the included studies did not report the amount of parenchyma above the lesion, which may cause some heterogeneity in the results.

## 5 Conclusions

Our study confirms that while CERT is associated with a greater decline in eGFR and a lower rate of trifecta achievement, its perioperative, functional, and oncological outcomes are comparable to non-CERT in RAPN. In centers with appropriate robotic surgical expertise, RAPN can be considered a minimally invasive surgical treatment for these lesions. However, to strengthen the evidence base and affirm the veracity of the findings, further extensive and meticulous research is indispensable, encompassing a larger sample size and comprehensive data from high-volume medical centers.

## Data Availability

The original contributions presented in the study are included in the article/**Supplementary Material**. Further inquiries can be directed to the corresponding author.
