# Supplementary material for: Impacts of completely endophytic renal masses on perioperative, oncologic, and functional outcomes in robot-assisted partial nephrectomy: a systematic review and meta-analysis
Source: Front Oncol. 2024 Oct 25;14:1444477. doi: 10.3389/fonc.2024.1444477 (PMC11543353; doi:10.3389/fonc.2024.1444477)
Supplement: Supplementary file 1 [file Table1.docx]

| **Table S1 The risk of bias (Non-RCTs)-ROBINS-I** | | | | | | |  |
| --- | --- | --- | --- | --- | --- | --- | --- |
| Bias domain | Ito | Motoyama | Carbonara | Curtiss | Komninos | Autorino |  |
|  |  |  |  |  |  |  |  |
| Bias due to confounding | Moderate | Moderate | Moderate | Moderate | Moderate | Moderate |  |
|  |  |  |  |  |  |  |  |
| Bias in selection of participants into the study | Low | Low | Low | Moderate | Low | Moderate |  |
|  |  |  |  |  |  |  |  |
| Bias in classification of interventions | Moderate | Moderate | Low | Moderate | Low | Low |  |
|  |  |  |  |  |  |  |  |
| Bias due to deviations from intended interventions | Low | Moderate | Moderate | High | Low | Moderate |  |
|  |  |  |  |  |  |  |  |
| Bias due to missing data | Moderate | Low | Low | Low | Moderate | Low |  |
|  |  |  |  |  |  |  |  |
| Bias in measurement of outcomes | Low | Moderate | Low | Moderate | Moderate | Low |  |
|  |  |  |  |  |  |  |  |
| Bias in selection of the reported result | Moderate | Moderate | Moderate | Moderate | Moderate | Moderate |  |
|  |  |  |  |  |  |  |  |
| Overall bias | Moderate | Moderate | Moderate | High | Moderate | Moderate |  |
|  |  |  |  |  |  |  |  |
